# Supplementary material for: Under 10 mortality patterns, risk factors, and mechanisms in low resource settings of Eastern Uganda: An analysis of event history demographic and verbal social autopsy data
Source: PLoS One. 2020 Jun 11;15(6):e0234573. doi: 10.1371/journal.pone.0234573 (PMC7289412; doi:10.1371/journal.pone.0234573)
Supplement: S1 File — (DOCX) [file pone.0234573.s001.docx]

**The life-table analysis approach**

The life table approach was used to estimate age-specific mortality patterns. For the life table computation, let $t$ be the interval time $x$, $x-n$, $I_{t}$– be the number of participants who are at risk during interval $t$, $D_{t}$– number of participants who die during interval t, $C_{\frac{t}{2}}$ – number of participants who are censored during interval $t$, $N_{t}^{*}$ - average number of participants at risk during interval $t$, $q_{t}$– proportion dying during interval $t$, $p_{t}$– proportion surviving interval $t$, $S_{t}$– the proportion surviving past interval $t$ ( cumulative survival probability).

$$q_{t}=\frac{D_{t}}{N_{t}^{*}} (1)$$

$$p_{t}= 1-q_{t} (2)$$

$$S_{t+1}=p_{t+1}*S_{t} (3)$$

$$N_{t}^{*}=I_{t}-C_{\frac{t}{2}} (4)$$

Note that, the calculation of $N_{t}^{*}$assumed that the events of interest are occurring at the end of the interval and the censored events are assumed to occur uniformly or evenly throughout the interval. Therefore, an adjustment on the initial number of participants for each time interval ($I_{t}$) was made to reflect the average number of participants at risk during the interval $t$. The Mortality rate at time $t$ in person-years is calculated as in equation 5.

$$M_{t}=\frac{q_{t}}{Person-years} (5)$$

The Confidence Interval (CI) at 5% level of significance is calculated as;

$$95\%CI=M_{t}\pm1.96S.E (6)$$

$$S.E=\sqrt{\frac{M_{t}}{I_{t}}}\left( 1-S_{t} \right) (7)$$

Where S.E is Standard Error.
