# Supplementary material for: Under 10 mortality patterns, risk factors, and mechanisms in low resource settings of Eastern Uganda: An analysis of event history demographic and verbal social autopsy data
Source: PLoS One. 2020 Jun 11;15(6):e0234573. doi: 10.1371/journal.pone.0234573 (PMC7289412; doi:10.1371/journal.pone.0234573)
Supplement: S1 Table — (DOCX) [file pone.0234573.s002.docx]

**S1 Table: Individual characteristics distribution for completed and imputed data using 2005-2015 birth registration dataset**

|  | **Complete** | | **Missing** | | **% Imputed** |
| --- | --- | --- | --- | --- | --- |
|  | **Freq.** | **%** | **Freq.** | **%** |  |
| ***Overall total*** | *22,385* | *100.0* | *-* | *-* | *-* |
| **Sex** |  |  |  |  |  |
| Male | 11,192 | 50.0 | - | - | - |
| Female | 11,193 | 50.0 | - | - | - |
| ***Total*** | *22,385* | *100.0* | *0* | *0.0* | - |
| **Birth Category** |  |  |  |  |  |
| Singleton | 21,599 | 97.5 | - | - | 97.5 |
| Multiple | 564 | 2.5 | - | - | 2.5 |
| ***Total*** | *22,163* | *100.0* | *222* | *1.0* | *100.0* |
| **Birth weight** |  |  |  |  |  |
| >=2.5 Kg | 7004 | 86.9 | - | - | 85.5 |
| Low birth weight | 1059 | 13.1 | - | - | 14.5 |
| ***Total*** | *8063* | *100.0* | *14,322* | *64.0* | *100.0* |
| **Place of residence** |  |  |  |  |  |
| Urban | 7461 | 33.3 | - | - | - |
| Rural | 14,924 | 66.7 | - | - | - |
| ***Total*** | *22,385* | *100.0* | *0* | *0.0* | *-* |
| **Maternal age ^a^** |  |  |  |  |  |
| <20 | 993 | 4.4 | - | - | - |
| 20-29 | 9737 | 43.5 | - | - | - |
| 30+ | 11,655 | 52.1 | - | - | - |
| ***Total*** | *22385* | *100.0* | *0* | *0.0* | *-* |
| **Marital status ^b^** |  |  |  |  |  |
| Not married | 2113 | 11.7 | - | - | 12.8 |
| Married | 15,902 | 88.3 | - | - | 87.2 |
| ***Total*** | *18,015* | *100.0* | *4,370* | *19.5* | *100.0* |
| **Education level** |  |  |  |  |  |
| None | 5931 | 26.5 | - | - | - |
| Primary | 10,856 | 48.5 | - | - | - |
| Post primary | 5598 | 25.0 | - | - | - |
| ***Total*** | *22,385* | *100.0* | *0* | *0.0* | *-* |
| **Wealth index ^c^** |  |  |  |  |  |
| Index 1-2 | 8650 | 49.8 | - | - | 47.2 |
| Index 3 | 3758 | 21.6 | - | - | 21.0 |
| Index 4-5 | 4974 | 28.6 | - | - | 31.8 |
| ***Total*** | *17,382* | *100.0* | *5003* | *22.3* | *100.0* |
| *^a^ – because of the possibility of non-linearity between age and childhood mortality a restricted cubic splines analysis was used to determine the age-group, from which the childhood mortality was a u-shaped with mortality being higher among those aged below 20 and 30+ years*  *^b^ – the married women included those who were both officially married and cohabiting while the un-married women included those staying alone, widowed and divorced or separated*  *^c^ – household wealth index was calculated through principal component analysis and the items included were electricity, cassette, radio, fan, television, telephone, bicycle, fridge, television, generator, bicycle, fridge, motor vehicle and motorcycle ownership, household floor structure, household wall and roof structures, type of fuel used for cooking* | | | | | |
