# Supplementary material for: Under 10 mortality patterns, risk factors, and mechanisms in low resource settings of Eastern Uganda: An analysis of event history demographic and verbal social autopsy data
Source: PLoS One. 2020 Jun 11;15(6):e0234573. doi: 10.1371/journal.pone.0234573 (PMC7289412; doi:10.1371/journal.pone.0234573)
Supplement: S2 Table — (DOCX) [file pone.0234573.s003.docx]

**S2 Table: Determinants of birth weight, wealth and marital status missingness in birth registration dataset**

|  | Low birth weight missing | | Wealth status missing | | Marital status missing | |
| --- | --- | --- | --- | --- | --- | --- |
|  | OR | P-value | OR | P-value | OR | P-value |
| **Place of birth** |  |  |  |  |  |  |
| Public health facilities | 1.00 |  |  |  |  |  |
| Community delivery | 2.28 | <0.001 | - | - | - | - |
| Private Clinics | 2.10 | <0.001 | - | - | - | - |
| **Place of residence** |  |  |  |  |  |  |
| Urban | 1.00 |  | 1.00 |  | 1.00 |  |
| Rural | 1.35 | <0.001 | 0.45 | <0.001 | 0.66 | <0.001 |
| **Time of child death** |  |  |  |  |  |  |
| Child died after day one |  |  | - | - | - | - |
| First day or stillbirth | 2.05 | <0.001 | - | - | - | - |
| **Education level** |  |  |  |  |  |  |
| None | 1.00 | - | 1.00 | - | - | - |
| Primary | 0.30 | <0.001 | 0.83 | 0.018 | - | - |
| Post-primary | 0.20 | <0.001 | 1.01 | 0.918 | - | - |
| **Age group (in years)** |  |  |  |  |  |  |
| <20 | 1.00 | - | 1.00 | - | 1.00 |  |
| 20-29 years | 1.17 | 0.032 | 0.80 | 0.036 | 0.51 | <0.001 |
| 30 and above | 2.01 | <0.001 | 0.42 | <0.001 | 0.20 | <0.001 |
| **Marital status** |  |  |  |  |  |  |
| Not married | - | - | 1.00 |  | - | - |
| Married | - | - | 1.57 | <0.001 | - | - |
| ***Note:*** *The birth weight missingness was strongly associated with the place of delivery, the time of death, place of residence, education level and maternal age. The wealth missingness was associated with the maternal age, marital status and education level. Marital status was associated with the place of residence and maternal age.* | | | | | | |
