# Supplementary material for: Under 10 mortality patterns, risk factors, and mechanisms in low resource settings of Eastern Uganda: An analysis of event history demographic and verbal social autopsy data
Source: PLoS One. 2020 Jun 11;15(6):e0234573. doi: 10.1371/journal.pone.0234573 (PMC7289412; doi:10.1371/journal.pone.0234573)
Supplement: S3 Table — (DOCX) [file pone.0234573.s004.docx]

**S3 Table: Maternal morbidities experienced in the last 3 months of pregnancy using 2005-2015 Iganga-Mayuge verbal autopsy data**

| **Maternal obstetric conditions** | **Percentage (n=844)** |
| --- | --- |
| Overall | 77.49 |
| Febrile illness | 44.08 |
| Severe Abdominal pain in the last trimester | 30.69 |
| Blurred vision in the last trimester | 21.68 |
| Smelly vaginal discharge in the last trimester | 14.45 |
| Vaginal bleeding in the last trimester | 13.86 |
| Pallor in the last trimester | 13.03 |
| Heart diseases in the last trimester | 12.68 |
| Puffy face in the last trimester | 9.24 |
| Shortness of breath in the last trimester | 8.53 |
| High blood pressure in the last trimester | 7.58 |
| Convulsion during pregnancy | 4.74 |
| Epilepsy | 1.30 |
| Diabetes | 0.47 |
