# Supplementary material for: Under 10 mortality patterns, risk factors, and mechanisms in low resource settings of Eastern Uganda: An analysis of event history demographic and verbal social autopsy data
Source: PLoS One. 2020 Jun 11;15(6):e0234573. doi: 10.1371/journal.pone.0234573 (PMC7289412; doi:10.1371/journal.pone.0234573)
Supplement: Attachment — Submitted filename: review comments.docx [file pone.0234573.s005.docx]

Overall

The topic itself is very interesting and contains meaningful insight, which should to be shared with the global health community. The manuscript deserves to be published; however, it needs to be revised.

The authors seem to have included too much information, particularly in the results and discussion session. I suggest the authors selecting only one or two study aim(s) and align the results and discussion with the study aim(s).

In addition, I suggest them comparing existing studies relating to under-five child mortality. In particular, the majority of the previous studies have been focusing on under-five child deaths or child mortality. If the authors are selling the potential readers on the under-10 child mortality, they need to make the manuscript more attractive by comparing their findings with the existing studies on the under-five child deaths. Also, I suggest they add at least one paragraph explaining current global strategy to tackle child mortality by applying life-course approach since they are also emphasizing the importance of the approach. I introduced some references they might refer to as below.

Major comments

(line 46-47) (line 60-61), (line 73-74) and many other paragraphs.

The authors seem to have described too many study aims. Can they clarify their study aims by streamlining these into just one or two specific aim(s)?

I understand that the authors aim to explore the causes and risk factors of children aged 5-9 years. They need to describe the reason why it is important to understand the causes and risk factors of this age group (5-9 years of children).

Based on the results session in the manuscript, the under-10 and 5-10 mortality probabilities are 129 and 11 per 1000 live births, which means that 5-10 age group takes only 8.5% of deaths among the children under 10 years. It may sound to some readers that this study just re-affirms the critical importance of focusing on under-five child mortality. Therefore, the authors need to explain the reasons why we still should pay attention to the mortality of this age group in spite of the small proportion of child deaths compared to the under-5 age group.

This will make the manuscript more attractive.

It would be good if the authors could compare their results with existing literature particularly exploring (1) global, regional, and national under-five child mortality (2) global, regional, and national mortality in older children (3) global, regional, and national cause-specific under-five child mortality.

The authors need to cite more up-to-date references and also a sort of global, regional and national child mortality studies needs to be referenced particularly for the under-five child mortality, risk factors, and cause-specific child deaths. For example, please cite the following articles.

-Global, regional, and national causes of under-5 mortality in 2000–15: an updated systematic analysis with implications for the Sustainable Development Goals (Li Liu, Shefali Oza, Dan Hogan, Yue Chu, Jamie Perin, Jun Zhu, Joy E Lawn, Simon Cousens, Colin Mathers, Robert E Black)

-Global, regional, and national under-5 mortality, adult mortality, age-specific mortality, and life expectancy, 1970-2016: a systematic analysis for the Global Burden of Disease Study 2016.

- Global, regional, and national comparative risk assessment of 84 behavioural, environmental and occupational, and metabolic risks or clusters of risks for 195 countries and territories, 1990–2017: a systematic analysis for the Global Burden of Disease Study 2017

Since the authors highlight the life-course approach for child or maternal health intervention, I think it is also good for them to cite the key reports and world strategy in child and maternal health emphasizing the life-course approach as follows:

-Global strategy for women’s, children’s and adolescents’ health (2016-2030)

-Global strategy for women’s, children’s and adolescents’ health (2016-2030): 2018 monitoring report (<https://www.who.int/pmnch/activities/advocacy/globalstrategy/2016_2030/commitments-report/en/>)

- Partnership for maternal, newborn & child health report (<https://www.everywomaneverychild.org/>)

- Countdown to 2030 report (<https://www.countdown2030.org/>)

The authors need to reflect the continuum of care in existing literature. Continuum of care is a representative concept for providing health care service along the life course. It would be good if the authors make their arguments citing the existing studies about this.

-Please cite this article. “Continuum of care for maternal, newborn, and child health:

from slogan to service delivery (Kate J Kerber, Joseph E de Graft-Johnson, Zulfi qar A Bhutta, Pius Okong, Ann Starrs, Joy E Lawn)

The authors need to follow the PLOS One authors guideline. In particular, the reference style in the manuscript is not the one for the PLOS One.

The title needs to be revised.

I suggest that the authors replace “in low resource settings” with “eastern and central parts of Uganda” or “Iganga-Mayuge Province (Please check whether “Province” is correct.) of Uganda” in the title.

Also, “causes and risk factors” seem to be more appropriate than “mortality patterns and risk factors”.

Again, isn’t it more appropriate to separate the “under 10 mortality” in the title into “under-five children” and “children aged 5-10 years”?

Minor comments

(line 49-51)

I think that it is not specific to the 5-9 years of age group.

(line 52-53)

Did the authors want to emphasize the importance of infectious disease even among those aged 5-9 years?

Throughout the manuscripts, “per 1000” needs to be replaced with “per 1000 live births”.

(line 85-92)

Can the authors add the following information to the current paragraph?

- how the HDSS selected households (i.e. sampling methods)

- target population (i.e. total population size in Iganga and Mayuge; what is the administrative unit of “Iganaga and Mayuge”?; basic demographic information of the population in Iganga and Mayuge; etc.)

- Why authors did choose this area for their study?

(line 117) Can the authors share the standard operating procedures as a Supplementary Information?

The authors need to proofread the manuscript more carefully. For instance, line 137 “The managed and analyzed in STATA…”

(line 178-180) Can the authors explain why they interpreted that “it indicates a possibility of birth weight missingness at random?” The Table 2 seems to show that there might be a sort of some tendency in the missing proportion. For example, the proportion of missing is higher in community delivery; rural areas; first day or still birth; no education. Except “the age group”, I think the other variables may indicate seemingly more vulnerable group. My interpretation might be wrong. I just suggest the authors add their interpretation to the current paragraph.

(line 196-202) Usually, study limitations are to be presented in the discussion sessions as the authors already knew, I guess. If the authors keep the paragraph relating to the limitation in the methods session, I suggest they rephrase the current one. For example, “because of the limitation in …, we restricted our analysis to….”

(line 211-216) If under-10 mortality probability was 129 and 5-10 was 11 per 1000, respectively, shouldn’t the under-five child mortality be 118 deaths per 1000 live birth? How could the authors present 100 per 1000 livebirths for under-five mortality probability?

(line 240) “the post-neonatal, child and 5-9 years of age respectively”. In this sentence, how did you define “child?” Is it a typo?

(line 238-242) Many existing studies including the WHO health statistics, or global/regional/national under-five studies do not consider mal-nutrition such as protein deficiency or any other nutritional causes as a “direct” cause of child death. The authors may check existing studies which I suggested as a reference above, malnutrition is being dealt with as an indirect cause of child deaths. Also, there seems to be some difference in cause-specific under-five child deaths between the results and existing studies. Can the authors explaining their results in comparison with the previous studies (global/regional/ and national under-five child deaths) in the discussion session?
