# Supplementary material for: Under 10 mortality patterns, risk factors, and mechanisms in low resource settings of Eastern Uganda: An analysis of event history demographic and verbal social autopsy data
Source: PLoS One. 2020 Jun 11;15(6):e0234573. doi: 10.1371/journal.pone.0234573 (PMC7289412; doi:10.1371/journal.pone.0234573)
Supplement: Attachment — Submitted filename: Response to Reviewers.docx [file pone.0234573.s006.docx]

**Reviewers comments**

**Reviewer: 1**

|  | **Responses** |
| --- | --- |
| Reviewer #1: This is an interesting paper exploring under 10 mortality patterns, risk factors and mechanisms in low resource settings in Uganda. The authors have done a careful analysis and methods and results are clearly presented. There is a good discussion of results and conclusions are supported by the results. However, there are some questions and suggestions I would have on my end: | *Thank you for such encouraging statements.* |
| 1. The paper is part of a doctoral thesis work. It still includes some details in methods and results that are not usually reported in scientific paper, although they are crucial in a thesis. Compressing the paper would facility its dissemination and understanding. For example, the paper now has 7 tables and 6 figures, unusually long for a scientific paper. I would suggest to select only the key results for the body of the text and move some tables and figures to additional files. the methods section can be simplified as well. | *Thanks for pointing this out. We have moved some of the information in the methods and results section including the tables as supplements (S1 File and S1 Table-S3 Table).* |
| 2. The authors mentioned VAs were used in the study, although unfortunately it was not possible to link their results with survey data to explore risk factors by cause of death. Anyway, the authors mentioned the cause of death was defined from a physician review (2) of the VA. Didn´they consider the use of any of the automated methods available now (Insylico, SmartVA?) It would be good to have more details on the method of review of the VA in the methods sections. it is only briefly mentioned, and details are actually provided in the discussion. | *Thanks for pointing this out. The analysis of the VA data depended on the final causes of death that was provided in the shared dataset. Indeed, as the reviewer points out, the best approach would have been using the* *Insylico or SmartVA if the whole dataset with all the VA questions or variables was provided. It would have actually helped us to validate the codes assigned by the Physicians. Perhaps this could be another project we could work on by engaging the HDSS to provide the dataset on VA will all variables.*  *We have included a sentence in the methods section highlighting why we could not use the software for assigning the causes of death code, as “Additionally, the dataset did not include all the variables that could be used to assign the causes of death using the available software algorithm such as InSilicoVA (McCormick et al., 2016) or SmartVA (Serina et al., 2015) or InterVA (Byass et al., 2019) and thus, the results only depend on the causes of death that were assigned by the Physicians”.* – **page 9, Lines 193-197** |
| 3. The authors used multiplie imputations to deal with the high missingnes found in the database. In page 9, line 178, it says "The place of delivery... and maternal age were associated with the birth weight missigness- indicating a possibility of birth weight missingness at random". If missingness is associated with some variables, how come it can be at random? I don´t think so. In this case, did the imputation method used is suitable for non-random missingness? | *Thank you for pointing this out. There are different missingness mechanisms. 1) Missingness Completely at Random – where a variable is missing completely at random if the probability of missingness is the same for all units, and 2) Missingness at random - this is when the probability a variable is missing depends only on available information. For instance, in our study, we assume that the place of delivery, the time of death as complete records affect LBW missingness. We, therefore, believe that given these assumptions, our imputation approach was appropriate.*  *We have included more information on imputation to guide the readers as “Our consideration of missing at random is based on the assumption that the missingness of variables’ fields depends on some of the observable variables (Carpenter & Kenward, 2013; Pampaka, Hutcheson, & Williams, 2016)”.* ***Page 8, Line 177-178***  *We also indicate that the imputation results are reliable as “There were modest differences between the imputed and complete discriptive statitics for variables with missing field (S1 Table).* ***Page 8, Line 183-184*** |
| 4. As the authors mentioned, there are not that many studies of under 10 mortality. The authors make a good case showing differences in causes and levels of death in some ages within this group (e.g. under vs. over 5). However, they need to make a strong case of why it is important to analyze this group, and what is the contribution of this study. These points are mentioned in the discussion, but authors could elaborate more on this. | *Thank you for pointing this out. We have included a sentence on the need for studying those aged 5+ in the discussion section as “Particularly, our study provides under-10 and 5-9 years mortality estimates and the importance of HDSS data in mortality estimates in areas with no or weak vital registration systems. Thus, our results contribute to the much-needed accountability of how children, particularly in a low resource's context, survive at each critical stage of development”.* **Page 16, Line 294-298**  And introduction section section as *“*I*n line with the current global Sustainable Development Goal (SDG) theme of “leaving no one behind” [19], United Nations’ Secretary-General's Health Global Strategy for Women, children and adolescents [20], and the WHO–UNICEF–Lancet Commission on children [7], evidence that accounts for when and why do children die before their 10^th^ birth day is an initial step for generating optimal solutions that may accelerate the SDG global and national commitments”.* **Page 4, Line 86-90**  *And “Whilst there is global enormous evidence on under-five mortality rate[1,2], very little on the level, patterns and mortality causes among 5-10 age group is known as the group is inadequately included in global reporting, yet in countries with high mortality rates, children aged 5-10 may remain at high risk of morbidity and mortality”,* **Page 3, Line 52-55** |

**Reviewer: 2**

|  | **Responses** |
| --- | --- |
|  |  |
| Overall  The topic itself is very interesting and contains meaningful insight, which should to be shared with the global health community. The manuscript deserves to be published; however, it needs to be revised. | *We are appreciative of the reviewer’s comment on the global contribution of this study.* |
| The authors seem to have included too much information, particularly in the results and discussion session. I suggest the authors selecting only one or two study aim(s) and align the results and discussion with the study aim(s). | *We have looked at the objectives as suggested by the reviewer and we think we should leave them the way they are to make it different from what is already in the publication space. These objectives are interrelated and we intended to use them to describe the child survival system. However, to reduce the length of the manuscript, we have transferred some of the information in the methods and results sections to the supplementary document.* |
| In addition, I suggest them comparing existing studies relating to under-five child mortality. In particular, the majority of the previous studies have been focusing on under-five child deaths or child mortality. If the authors are selling the potential readers on the under-10 child mortality, they need to make the manuscript more attractive by comparing their findings with the existing studies on the under-five child deaths. Also, I suggest they add at least one paragraph explaining current global strategy to tackle child mortality by applying life-course approach since they are also emphasizing the importance of the approach. I introduced some references they might refer to as below. | *Thanks to the reviewer for providing us with more evidence to include in our manuscript. We have considered all of them appropriately. These include 3, 4, 6, 7, 19, 20, 21, and 22.*  *We have also included a statement on under-5 mortality at the beginning of the introduction as “Whilst there is global enormous evidence on under-five mortality rate,(United Nations Inter-agency Group for Child Mortality Estimation (UN IGME), 2018, 2019) very little on the level, patterns and mortality causes among 5-10 age group is known as the group is inadequately included in global reporting, yet in countries with high mortality rates, children aged 5-10 may remain at high risk of morbidity and mortality”.* **Page 3, Line 52-55**  *We have also added a paragraph on global strategies as “In line with the current global Sustainable Development Goal (SDG) theme of “leaving no one behind” , United Nations’ Secretary-General's Health Global Strategy for Women, children and adolescents [19], and the WHO–UNICEF–Lancet Commission on children [7], there is a need for evidence that accounts for when and why do children die before their 10^th^ birth day if optimal solutions to accelerate the SDG global and national commitments are to be generated”.* **Page 4, Line 86-90** |
| **Major comments**  (line 46-47) (line 60-61), (line 73-74) and many other paragraphs.  The authors seem to have described too many study aims. Can they clarify their study aims by streamlining these into just one or two specific aim(s)? | *We have looked at the objectives as suggested by the reviewer and we think we should leave them the way they are to make it different from what is already in the publication space. These objectives are interrelated and we intended to use them to describe the child survival system and the need for a life-cource approach. However, to reduce the length of the manuscript, we have transferred some of the information in the methods and results sections as supplementary files (S1 File and S1-S3 Tables).* |
| I understand that the authors aim to explore the causes and risk factors of children aged 5-9 years. They need to describe the reason why it is important to understand the causes and risk factors of this age group (5-9 years of children). Based on the results session in the manuscript, the under-10 and 5-10 mortality probabilities are 129 and 11 per 1000 live births, which means that 5-10 age group takes only 8.5% of deaths among the children under 10 years. It may sound to some readers that this study just re-affirms the critical importance of focusing on under-five child mortality. Therefore, the authors need to explain the reasons why we still should pay attention to the mortality of this age group in spite of the small proportion of child deaths compared to the under-5 age group.  This will make the manuscript more attractive. | *Thank you for pointing this out. We have included a sentence on the need for studying those aged 5+ in the discussion section as “Particularly, our study provides under-10 and 5-9 years mortality estimates and the importance of HDSS data in mortality estimates in areas with no or weak vital registration systems. Thus, our results contribute to the much-needed accountability of how children, particularly in a low resource's context, survive at each critical stage of development”.* **Page 16, Line 294-298**  *We have included the need for the study re-aligning it with the global strategies “In line with the current global Sustainable Development Goal (SDG) theme of “leaving no one behind” , United Nations’ Secretary-General's Health Global Strategy for Women, children and adolescents [19], and the WHO–UNICEF–Lancet Commission on children [7], there is a need for evidence that accounts for when and why do children die before their 10^th^ birth day if optimal solutions to accelerate the SDG global and national commitments are to be generated”* **Page 4, Line 86-90** *and the inadquaces in evidence for those aged 5-10 years “Whilst there is global enormous evidence on under-five mortality rate,(United Nations Inter-agency Group for Child Mortality Estimation (UN IGME), 2018, 2019) very little on the level, patterns and mortality causes among 5-10 age group is known as the group is inadequately included in global reporting, yet in countries with high mortality rates, children aged 5-10 may remain at high risk of morbidity and mortality”.* **Page 3, Line 53-55** |
| It would be good if the authors could compare their results with existing literature particularly exploring (1) global, regional, and national under-five child mortality (2) global, regional, and national mortality in older children (3) global, regional, and national cause-specific under-five child mortality.    The authors need to cite more up-to-date references and also a sort of global, regional and national child mortality studies needs to be referenced particularly for the under-five child mortality, risk factors, and cause-specific child deaths. For example, please cite the following articles. | *Thanks to the reviewer for providing us with more evidence to include in our manuscript. We have considered all of them appropriately. These include 3, 4, 6, 7, 19, 20, 21, and 22.* |
| The authors need to reflect the continuum of care in existing literature. Continuum of care is a representative concept for providing health care service along the life course. It would be good if the authors make their arguments citing the existing studies about this.  -Please cite this article. “Continuum of care for maternal, newborn, and child health:  from slogan to service delivery (Kate J Kerber, Joseph E de Graft-Johnson, Zulfi qar A Bhutta, Pius Okong, Ann Starrs, Joy E Lawn) |  |
| The authors need to follow the PLOS One authors guideline. In particular, the reference style in the manuscript is not the one for the PLOS One. | *Thank you for pointing this out. We have now changed the reference style to that of PLOS ONE* |
| The title needs to be revised.  I suggest that the authors replace “in low resource settings” with “eastern and central parts of Uganda” or “Iganga-Mayuge Province (Please check whether “Province” is correct.) of Uganda” in the title.  Also, “causes and risk factors” seem to be more appropriate than “mortality patterns and risk factors”.  Again, isn’t it more appropriate to separate the “under 10 mortality” in the title into “under-five children” and “children aged 5-10 years”? | *Thank you for the advice. We have revised the title considering your comments. It now reads “**Under 10 mortality patterns, risk factors, and mechanisms in low resource settings of Eastern Uganda: an analysis of event history demographic and verbal social autopsy data”*  *We have maintained mortality patterns since one of our objectives is to study the mortality estimates by age-groups.* |
| **Minor comments**  (line 49-51)  I think that it is not specific to the 5-9 years of age group.  (line 52-53)  Did the authors want to emphasize the importance of infectious disease even among those aged 5-9 years?  Throughout the manuscripts, “per 1000” needs to be replaced with “per 1000 live births”. | *We have edited it to make it clearer. The known infections such as malaria, pneumonia and diarrhoea that occur among under-5 may affect even those aged 5-9 years.*  *Per 1000 live birth applies to only those whose denominator considers all the birth, for instance, under-5 and under-10 and may not apply for 5-9 years since the denominator considers only those who survived up to 5^th^ birthday. We have now considered it as per 1000 children aged 5-9 years* |
| (line 85-92)  Can the authors add the following information to the current paragraph?  - how the HDSS selected households (i.e. sampling methods)  - target population (i.e. total population size in Iganga and Mayuge; what is the administrative unit of “Iganaga and Mayuge”?; basic demographic information of the population in Iganga and Mayuge; etc.)  - Why authors did choose this area for their study?  (line 117) Can the authors share the standard operating procedures as a Supplementary Information? | *Thank you for pointing this out. We have included the table (Table 1, Page 5) summarizing the household and population for the two districts indicating the subcounties covered by the HDSS.*  *The HDSS uses the INDEPTH/WHO tools which are publically available on the website. We have cited the INDEPTH website with the page location of the tools.*  INDEPTH Network. (n.d.). The Verbal and Social Autospy tools. Retrieved May 8, 2020, from <http://www.indepth-network.org/resources/tools> |
| The authors need to proofread the manuscript more carefully. For instance, line 137 “The managed and analyzed in STATA…” | *Thank you for pointing this out. We have read the manuscript for all typos.* |
| (line 178-180) Can the authors explain why they interpreted that “it indicates a possibility of birth weight missingness at random?” The Table 2 seems to show that there might be a sort of some tendency in the missing proportion. For example, the proportion of missing is higher in community delivery; rural areas; first day or still birth; no education. Except “the age group”, I think the other variables may indicate seemingly more vulnerable group. My interpretation might be wrong. I just suggest the authors add their interpretation to the current paragraph. | *We agree with the reviewer’s observation on factors associated with missingness, however, since the paper’s focus is not multiple imputations, we may not need to spend time to discuss why such factors could be associated with LBW missingness. Perhaps, it is something else we shall have to consider for our future research projects.*  *We have included a sentence to support the conclusion of missing at random with some references as “Our consideration of missing at random is based on the assumption that the missingness of variables’ fields depends on some of the observable variables (Carpenter & Kenward, 2013; Pampaka, Hutcheson, & Williams, 2016).”* ***Page 8, Line 177-178*** |
| (line 196-202) Usually, study limitations are to be presented in the discussion sessions as the authors already knew, I guess. If the authors keep the paragraph relating to the limitation in the methods session, I suggest they rephrase the current one. For example, “because of the limitation in …, we restricted our analysis to….” | *We appreciate the reviewer's suggestion. However, we have maintained the way it was written as the first reviewer had a comment on why we never used the available computer algorithm.* |
| (line 211-216) If under-10 mortality probability was 129 and 5-10 was 11 per 1000, respectively, shouldn’t the under-five child mortality be 118 deaths per 1000 live birth? How could the authors present 100 per 1000 livebirths for under-five mortality probability? | *We understand the reviewer’s concern. However, the results should be interpreted as probabilities as calculated using life-tables but not rates and there are in most cases miss-interpreted or proportions of death within the interval – we have the note in table 1 to help the readers with the interpretation. We also provided the supplementary file on the life-table mortality estimates and how they were applied in this study. Thus, it is wrong to add up the age-specific probabilities to generate a cumulative probability for the overall group.*  *We also share some writeup on motarity probabilities and rates in the link below.*  <https://www.who.int/whosis/whostat2006InfantAndUnder5MortalityRate.pdf> |
| (line 240) “the post-neonatal, child and 5-9 years of age respectively”. In this sentence, how did you define “child?” Is it a typo? | *We meant child mortality to be those aged 1-4 years. we have now specified the actual age band for clarity as “1-12 months (post-neonatal), 1-4 years and 5-9 years of age”.* |
| (line 238-242) Many existing studies including the WHO health statistics, or global/regional/national under-five studies do not consider mal-nutrition such as protein deficiency or any other nutritional causes as a “direct” cause of child death. The authors may check existing studies which I suggested as a reference above, malnutrition is being dealt with as an indirect cause of child deaths. Also, there seems to be some difference in cause-specific under-five child deaths between the results and existing studies. Can the authors explaining their results in comparison with the previous studies (global/regional/ and national under-five child deaths) in the discussion session? | *You have raised an important issue around the verbal autopsy, where we depend on the final codes assigned by the physicians. The causes of death are assigned using the ICD-10 guidelines and malnutrition is a group that is described by E40–E46 codes in the ICD-10. Our analysis depends on the final codes assigned by the physicians and malnutrition was classified as a cause of death.*  *In the study context, the prevalence of malnutrition is reported to be high and we think having it reported as it was assigned by the physicians would bring insight into how the other neglected mortality causes of the poor such as malnutrition could be emerging.* |
